# Effects of *Clostridium tyrobutyricum* on Lipid Metabolism, Intestinal Barrier Function, and Gut Microbiota in Obese Mice Induced by High-Fat Diet

**DOI:** 10.3390/nu16040493

**Published:** 2024-02-08

**Authors:** Yanqiu Luo, Yuyue Jin, Haidong Wang, Geng Wang, Yueying Lin, Haohan Chen, Xinyu Li, Minqi Wang

**Affiliations:** Key Laboratory of Molecular Animal Nutrition, Ministry of Education, College of Animal Sciences, Zhejiang University, Hangzhou 310058, China; 22117058@zju.edu.cn (Y.L.); jinyuyue@zju.edu.cn (Y.J.); wanghaidong@zju.edu.cn (H.W.); wanggeng@zju.edu.cn (G.W.); 22017011@zju.edu.cn (Y.L.); 22017099@zju.edu.cn (H.C.); lixinyu1502@126.com (X.L.)

**Keywords:** obesity, *C. tyrobutyricum*, lipid metabolism, intestinal barrier, intestinal inflammatory, short-chain fatty acids, cecal microbiota

## Abstract

Obesity and its complications constitute a main threat to global human health. The purpose of this investigation was to explore the influences of *Clostridium tyrobutyricum* (Ct) on lipid metabolism, intestinal barrier function, and intestinal microbiome in obese mice induced by a high-fat diet (HFD). After establishing the obesity model, 10^7^ CFU/mL and 10^8^ CFU/mL *C. tyrobutyricum* were used to intervene in HFD-fed mice by gavage for six weeks, and indexes related to obesity were measured. In the liver of HFD-fed mice, the results revealed that *C. tyrobutyricum* reduced liver weight and the levels of triglyceride (TG), total cholesterol (TC), and nonesterified fatty acid (NEFA), along with decreasing red lipid droplets and fat vacuoles. After *C. tyrobutyricum* intervention, the mRNA expression of peroxisome proliferator-activated receptor-γ (PPARγ) was downregulated, and AMP-activated protein kinase (AMPK), peroxisome proliferator-activated receptor-α (PPARα), adipose triglyceride lipase (ATGL), and hormone-sensitive lipase (HSL) were upregulated in the liver. Additionally, *C. tyrobutyricum* alleviated intestinal morphology injury caused by HFD, decreased the expression of tumor necrosis factor-α (TNF-α), interleukin 6 (IL-6), and IL-1β in the colon, and upregulated tight junction protein expression. In addition, 16S rRNA sequencing revealed that *C. tyrobutyricum* increases the diversity of intestinal microbiota. Overall, *C. tyrobutyricum* improved HFD-induced lipid metabolism disorders, preserved the intestinal barrier’s integrity, and modulated the structure of the intestinal microbiome. These findings provide a novel insight into the role of *C. tyrobutyricum* as a probiotic in regulating lipid metabolism.

## 1. Introduction

In recent years, the rapidly growing epidemic of obesity, which is a metabolic disease, has become a threat that undermines the future global health progress [1]. As reported by the Lancet, between 1975 and 2016, the age-standardized incidence of obesity increased globally, rising from 0.9% to 7.8% in boys and from 0.7% to 5.6% in girls [2]. Obesity is mostly caused by an imbalance between energy intake and consumption [3]. It can typically cause endocrine disorders as well as hypercholesterolemia, hyperlipidemia, and hypertension, all of which in turn raise the chance of developing cardiovascular disease, type 2 diabetes, and several cancers [4,5,6,7,8]. Astonishingly, the direct healthcare costs and lost economic productivity of obesity are estimated at about USD 2 trillion annually [9]. Thus, effective responses for its prevention are required. Currently, the choice of therapeutic interventions for obesity is multi-modality, including combinations of lifestyle adjustment, weight loss drugs, and bariatric surgery [10,11].

Fatty acid transport, synthesis, and oxidation are critical steps in lipid metabolism that rely on the regulation of several transcription factors, and obesity is linked to these processes. In adipocytes, one potential target for dissipating fat stores and remodeling adipocyte lipid and glucose metabolism is AMP-activated protein kinase (AMPK) [12]. Triacylglycerol turnover and lipid partitioning are regulated by the vital hepatic lipase adipose triglyceride lipase (ATGL) [13]. The crucial enzyme that supplies glycerol and free fatty acids in the lipolysis process is hormone-sensitive lipase (HSL) [14]. Lipid sensors called peroxisome proliferator-activated receptors (PPARs) regulate the body’s overall energy metabolism. Among them, PPARα regulates the transport, oxidation, and ketogenesis of fatty acids. In comparison, PPARγ is a primary regulator of adipogenesis [15]. In addition, 1-amino-cyclopropane-1-carboxylic acid (ACC), as well as sterol regulatory element-binding protein 1c (SREBP1c), are central transcription activators of fatty acid biosynthesis [16,17]. Accordingly, controlling the mRNA expression levels of these genes is very important for tackling lipid metabolic disorders linked to obesity.

It has been hypothesized that the gut–liver axis is essential for the pathophysiology of obesity. An increased consumption of obesogenic foods may affect the intestinal barrier function and gut microbiota, thereby encouraging the development of obesity and fatty liver disease [18]. Accumulating evidence has highlighted the anti-obesity effect of probiotics. Treatment with *A. muciniphila* reversed fat mass growth and adipose tissue inflammation caused by HFD, as shown by Everard et al. [19]. Some probiotics connected to an anti-obesity effect, such as *Bifidobacterium* and *Lactobacillus*, were found to balance the obesity-related intestinal flora and modulate the inflammatory response [20]. *C. tyrobutyricum* (Ct) is a typical butyric acid-producing Gram-positive anaerobe. Emerging evidence suggests that Ct can modulate gut health and intestinal barrier function and have specific anti-inflammatory effects, such as improving the integrity of the uterine barrier and alleviating the colon inflammatory reaction [21,22]. Our earlier study has also shown that Ct may prevent the rise in intestinal permeability brought on by LPS and prevent intestinal apoptosis in IPEC-J2 cells [23].

To our knowledge, not much research has been carried out into how Ct supplementation affects lipid metabolism and its underlying mechanisms. Transcriptome sequencing performed by our laboratory revealed that Ct can reverse metabolic processes, including the synthesis and breakdown of fatty acids. As evidenced by our Kyoto Encyclopedia of Genes and Genomes results, the adipocytokine signaling pathway was enriched for carnitine palmitoyltransferase-1 and acetyl-CoA carboxylase 2 (ACC2) [24]. In other words, Ct can play an essential role in host health by modulating intestinal immunity and may participate in regulating lipid metabolism. We hypothesized that Ct could alleviate obesity by modulating intestinal health and alleviating the inflammatory reaction. Thus, the goal of this research was to delve into the consequences of different concentrations of Ct supplementation on the lipid metabolism, microbiota, and the function of intestinal barriers in a mouse model of HFD-induced obesity, ultimately identifying the possible utilization of Ct as a probiotic for alleviating obesity.

## 2. Materials and Methods

### 2.1. Mice and the Design of Experiments

The Zhejiang Academy of Medical Sciences supplied four-week-old C57BL/6J male mice for breeding at the Zhejiang University Laboratory Animal Center. All mice were housed in cages maintained at a 12 h light and dark cycle at 25 °C with ad libitum access to the distilled water. Zhejiang University’s Institutional Animal Care and Use Committee approved and governed all experimental protocols and animal care (approval date: 18 October 2022; approval number: ZJU20220389).

**Figure 1:**
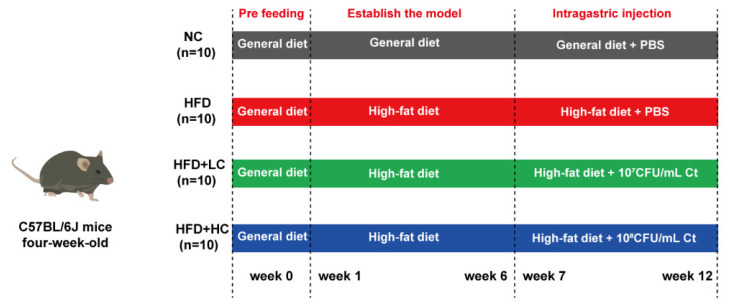
Schematic for the feeding experiment. Male C57BL/6J mice received the high-fat or general diet for six weeks to build the model. After that, the mice received phosphate-buffered saline (PBS), 10^7^ CFU/mL Ct, or 10^8^ CFU/mL Ct treatment by gavage in 0.2 mL volumes for six weeks.

As shown in Figure 1, the animals were first given the general diet alone for acclimation over the course of a week (4 to 5 weeks of age). At five weeks of age, the mice were randomized by body weight and assigned to their experimental diet groups. The control cohort was still sustained on the general diet (including fat (10 kcal%), protein (20 kcal%), and carbohydrate (70 kcal%), 3601 kcal/kg, *n* = 10). The other mice consumed the high-fat and high-calorie diet (including fat (60 kcal%), protein (20 kcal%), and carbohydrate (20 kcal%), 5128 kcal/kg, *n* = 36) to establish the obesity model. The nutritional composition of the general diet and the high-fat diet used throughout the study is shown in Table 1 (provider: Jiangsu Xietong Bioengineering Corporation, Nanjing, China).

The mice with BW that exceeded that of the control group by at least 20% were considered obese. After six weeks of chow diet or HFD feeding, a total of 30 obese mouse models were successfully established and randomly split into three experimental groups: HFD group, HFD + LC group, and HFD + HC group (*n* = 10 per group). The remaining six mice were removed from the study and euthanized due to the failure of modeling. For the following trial, eleven-week-old C57BL/6J mice received PBS (NC and HFD group, intragastric administration with PBS, *n* = 10 per group), low-dose Ct (HFD + LC group, intragastric administration with 10^7^CFU/mL Ct, *n* = 10), and high-dose Ct (HFD + HC group, intragastric administration with 10^8^CFU/mL Ct, *n* = 10) for six weeks. The control group was fed the control diet continuously during the intervention, while the high-fat diet was given to the other three groups.

The experiment ended when the blood was extracted from the retro-orbital plexuses at 17 weeks of age. After the mice were anesthetized with isoflurane, they were humanely euthanized by cervical dislocation. The liver was removed, and the wet weight of the liver was weighed. Subsequently, parts of the left liver lobe and intestinal tissues from the proximal colon, the proximal duodenum, the middle part of the jejunum, and the distal ileum were preserved at room temperature in a 4% paraformaldehyde solution for histological observation. The tissues that were left were snap-frozen and kept at −80 °C. The colonic content was gathered in order to measure SCFA. The formula for calculating the liver index was as follows: liver index (%) = liver wet weight/body weight × 100%.

### 2.2. Bacterial Culturing

Prof. Shang-Tian Yang (Ohio State University) kindly provided *C. tyrobutyricum* ATCC25755, and the Ct strain was cultured in accordance with our earlier research [25]. Briefly, Ct was cultivated anaerobically in *Clostridium* growth medium (CGM) at 37 °C followed by centrifuging for five minutes at 12,000× *g* at 4 °C. The bacteria were re-suspended after three washing steps with PBS. Finally, the Ct concentration was adjusted to 10^7^ and 10^8^ CFU/mL. The bacteria solution was freshly prepared before gavage every day.

### 2.3. Serum Biochemical and Hepatic Lipid Analyses

The activities of serum alanine aminotransferase (ALT) and aspartate aminotransferase (AST) were measured using the automatic biochemical analyzer (Olympus AU2700, Tokyo, Japan) at Affiliated Hospital of Hangzhou Normal University (Hangzhou, China). Total cholesterol (TC) (A111-1-1), triglyceride (TG) (A110-1-1), LDL cholesterol (LDL-C) (A113-1-1), and HDL cholesterol (HDL-C) (A112-1-1) in serum were measured using kits supplied by Nanjing Jiancheng Bioengineering Institute.

We weighed the livers and homogenized them in cold PBS (tissue weight (g): PBS volume (mL) = 1:9), subsequently centrifuging these for 15 min at 3500 rpm, and used the supernatant as the analytical sample. The concentrations of liver NEFA (A042-2-1), TG, and TC were determined using corresponding commercial diagnostic kits (Nanjing Jiancheng Bioengineering Institute, Nanjing, China).

### 2.4. Enzyme-Linked Immunosorbent Assay

The levels of IL-1β, IL-6, IL-10, and TNF-α in colon homogenates were measured with ELISA kits as per the guidelines (MLBIO Biotechnology, Shanghai, China).

### 2.5. Histological Investigation of Liver and Intestine

Briefly, 4% paraformaldehyde was used to fix the samples of the liver and intestine (colon, duodenum, jejunum, and ileum) for 24 h. Following fixation, the tissues underwent PBS rinsing, graded ethanol washes to remove moisture, and paraffin embedding. Sections (5 μm) were cut, and hematoxylin and eosin were used to stain them. A Nikon Eclipse 80i microscope (Tokyo, Japan) was used to take all the pictures related to intestine and liver morphology. In addition, the crypt depth and villus height were gauged using Caseviewer2.0 software. The colon’s histological damage was assessed and scored based on the criteria outlined in Table 2 [26].

After taking part of the tissues into the Tissue-Tek OCT compound, frozen liver slices (6 μm) were used for the oil red O staining procedure to visualize hepatic lipid accumulation.

### 2.6. Real-Time Quantitative PCR

The total RNA of the colonic and liver samples was extracted using the TRIzol reagent (Invitrogen, Carlsbad, CA, USA). The RNA concentration and purity were ascertained using a NanoDrop 2000 spectrophotometer (Thermo Fisher Scientific, Waltham, MA, USA). cDNA was produced with MonScript™ RTIII All-in-One Mix as directed by the manufacturer’s instructions (Monad, Wuhan, China). Real-time PCR was conducted with MonAmp™ SYBR^®^ Green qPCR Mix (Monad, Wuhan, China) via a CFX96™ Real-Time System (Bio-Rad, Hercules, CA, USA). Two steps were involved in the qPCR reaction procedure, and the following reaction conditions were established: thirty seconds at 95 °C, then forty cycles of 10 s at 95 °C and 30 s at 60 °C. β-actin was used as a housekeeping gene, and the method of 2^−ΔΔCt^ was employed to assess the relative mRNA expression. Table 3 and Table 4 display the PCR primers that were produced in Tsingke (Beijing, China).

### 2.7. Short-Chain Fatty Acids in Colonic Content

Gas chromatography (GC) was used to evaluate the short-chain fatty acid (SCFA) concentrations [27]. Absolute ethyl alcohol was mixed with the colon contents for 1 h. After 15 min of centrifugation at 10,000 rpm, the 85% orthophosphoric acid was added to the supernatant and left for 1 h. Subsequently, the supernatant liquor was filtered through microporous membranes with a pore size of 0.22 μm and transferred into the gas chromatography vial. GC was performed using a DB-624 capillary column (30 m × 0.32 mm × 1.8 μm, Agilent, Santa Clara, CA, USA) and flame ionization detector (FID).

### 2.8. Microbiome Sequencing and Analyses

The NC group, HFD group, and HFD + HC group were selected for analysis. From each group, six samples of the cecal contents were chosen at random to undergo high-throughput sequencing. That is, amplification and sequencing were performed on the bacterial 16S rRNA gene’s V3 + V4 hypervariable regions. Technical support for DNA extraction, PCR amplification, clone library construction, and sequencing was provided by Novogene Technology Co, Ltd. (Beijing, China).

### 2.9. Statistical Analysis

All data are presented with mean  ±  SEM, and all statistical analyses were performed with GraphPad Prism 8.0 software using one-way analysis of variance (ANOVA), followed by Tukey’s multiple comparison test. The statistical significance criterion was set at *p* < 0.05, with * *p* < 0.05 and ** *p* < 0.01 designating the significance level.

## 3. Results

### 3.1. Ct Inhibits HFD-Induced Body Weight Increase in Mice

**Figure 2:**
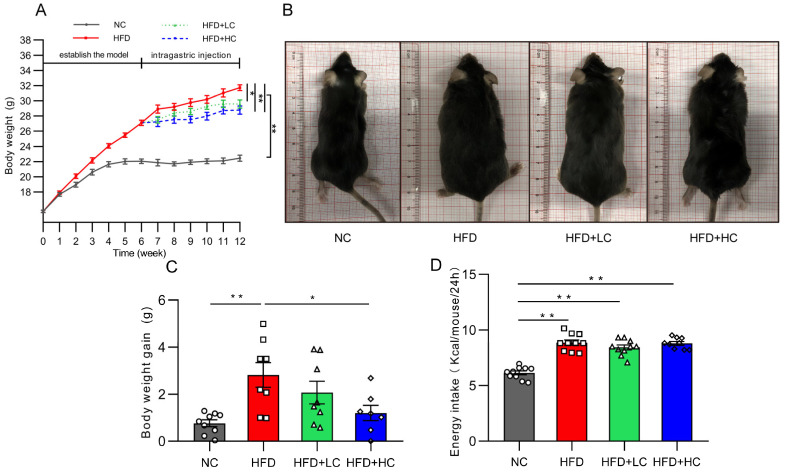
Impact of Ct on mice’s body weight and food intake. NC, mice administered general diet and gavaged with PBS; HFD, mice administered high-fat diet and gavaged with PBS; HFD + LC, mice administered high-fat diet and gavaged with 10^7^CFU/mL Ct; HFD + HC, mice administered high-fat diet and gavaged with 10^8^CFU/mL Ct. (**A**) Variations in body weight throughout the trial. The gray solid line represents the NC group, red solid line represents the HFD group, green dotted line represents the HFD + LC group, blue dotted line represents the HFD + HC group; (**B**) figures of mice body size at the end of the experiment; (**C**) weight gain during the administration period; (**D**) energy intake. The gray bars and open circles (○) represent the NC group, red bars and open squares (□) represent the HFD group, green bars and open triangles (△) represent the HFD + LC group, and blue bars and open rhombus (◇) represent the HFD + HC group. Data were analyzed using one-way ANOVA and expressed as mean ± SEM, *n* = 7–10. * *p* < 0.05 and ** *p* < 0.01.

At the initial stage, there was no discernible difference between the groups in the change in body mass index (*p* > 0.05, Figure 2A). As expected, HFD induced significant gains in body weight from baseline. Thirty obese mice were chosen for the follow-up intervention trial after six weeks of the HFD challenge, and their average body weight was 22.2% greater than that of the NC group. Figure 2A,C shows that the weight gain of the NC group and of the HFD + HC group was considerably reduced compared to that of the HFD group during the Ct intervention period (*p* < 0.05). The above results indicate that the high-fat diet can significantly induce obesity in mice, and *Ct* has a certain alleviation effect on obesity induced by the HF diet, while the effect of high-dose *Ct* intervention is more prominent.

Figure 2D presents data on energy intake. It can be noted that HFD mice actually had a higher energy intake compared with the NC group (*p* < 0.01). Nevertheless, no significant group variations were discovered in grams of food between groups fed the high-fat diet over the course of the study, suggesting that Ct’s mitigation of obesity was not associated with energy intake.

### 3.2. Ct Inhibits HFD-Induced Hyperlipidemia in Mice

**Figure 3:**
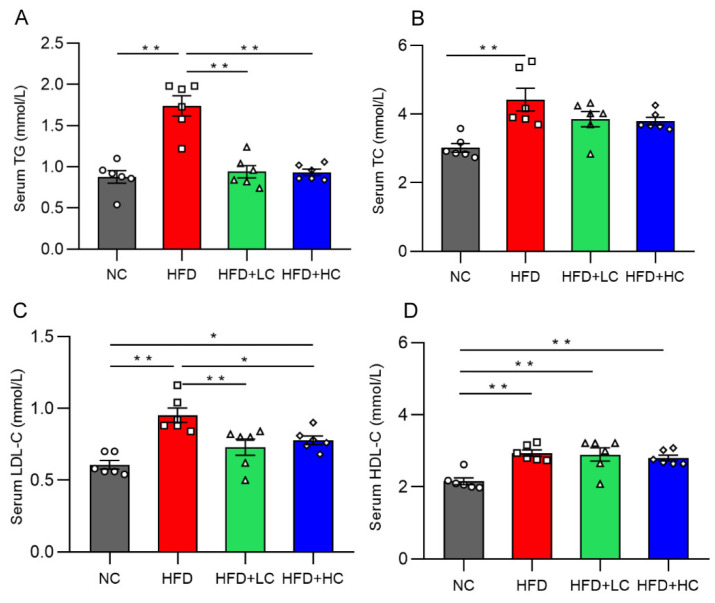
Effect of Ct on levels of serum lipid in mice at 17 weeks of age. (**A**) Serum TG; (**B**) serum TC; (**C**) serum LDL-C; (**D**) serum HDL-C. The gray bars and open circles (○) represent the NC group, red bars and open squares (□) represent the HFD group, green bars and open triangles (△) represent the HFD + LC group, and blue bars and open rhombus (◇) represent the HFD + HC group. Data are represented as mean ± SEM and were analyzed using one-way ANOVA, *n* = 6. * *p* < 0.05, ** *p* < 0.01.

Mice on the HF diet showed notably greater levels of TC, TG, HDL-C, and LDL-C compared to mice in the NC group (*p* < 0.01, Figure 3). In contrast, the TG and TC levels of the two treatment groups did not differ significantly from those of the NC group (*p* > 0.05, Figure 3A,B). In contrast to the HFD group, the TG and LDL-C content in the serum decreased significantly in the HFD + LC group by 45.92% and 23.42%. Meanwhile, the treatment with high-dose Ct markedly reduced the TG and LDL-C mean levels by 46.31% and 18.36%, respectively, as compared to the HFD group (*p* < 0.05, Figure 3A,C).

### 3.3. Effect of Ct on Liver Lipid Deposition in Obese Mice

**Figure 4:**
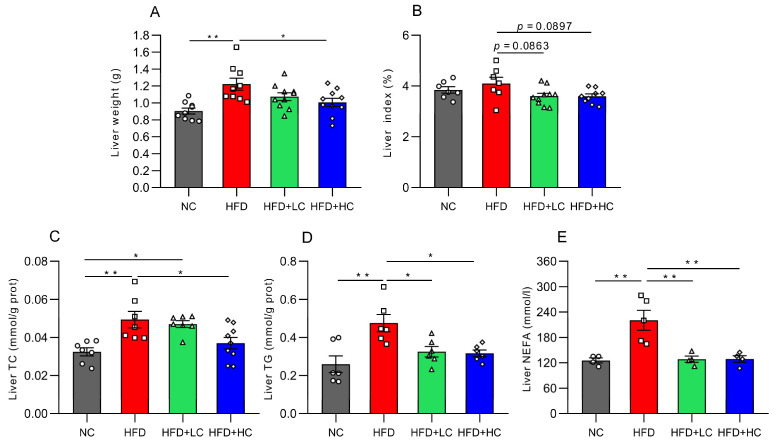
Impact of Ct on lipid accumulation in mice liver at 17 weeks of age. (**A**) Liver weight; (**B**) liver index; (**C**) liver TC level; (**D**) liver TG level; (**E**) liver NEFA level. The gray bars and open circles (○) represent the NC group, red bars and open squares (□) represent the HFD group, green bars and open triangles (△) represent the HFD + LC group, and blue bars and open rhombus (◇) represent the HFD + HC group. Data are represented as mean ± SEM and analysis with one-way ANOVA was performed, *n* = 4–10. * *p* < 0.05, ** *p* < 0.01.

Mice fed the high-fat diet for 12 weeks displayed significantly greater liver weights than mice on the regular chow diet (*p* < 0.01, Figure 4A), while 10^8^CFU/mL *C. tyrobutyricum* intervention was shown to markedly reduce liver weight (*p* < 0.05, Figure 4A). Moreover, both high- and low-dose groups exhibited decreasing trends in the liver index (*p =* 0.0897 and *p =* 0.0863, respectively, Figure 4B).

To learn more about the impacts of Ct on lipid metabolism in obese mice, the TC, TG, and NEFA levels were assayed. The results showed that these indices of the HFD group considerably grew in contrast to those in the NC group (*p* < 0.01, Figure 4C–E). Compared with the HFD group, this was corroborated by significant trends toward improvement in the liver’s TG level (31.67% reduction) and NEFA level (41.79% reduction) after Ct intervention at a concentration of 10^7^ CFU/mL (*p* < 0.05, Figure 4D,E). Meanwhile, in contrast to the HFD group, the TC, TG, and NEFA contents were reduced by 25.13%, 33.38%, and 41.52% in the 10^8^ CFU/mL Ct-treated group, respectively (*p* < 0.05, Figure 4C–E).

**Figure 5:**
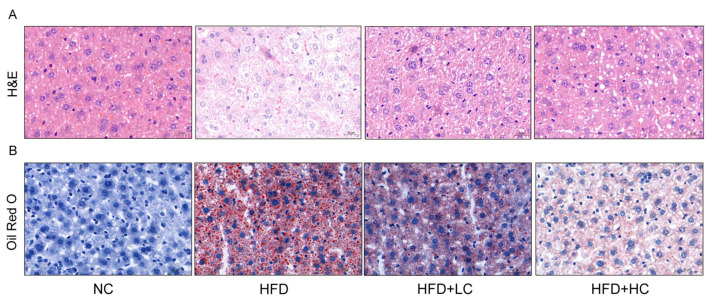
Effect of Ct on morphology changes in mice liver. (**A**) The picture of the liver section with H&E staining (scale bar: 20 μm); (**B**) the picture of the liver section with oil red O staining (scale bar: 50 μm).

Given that obesity is typically accompanied by hepatic steatosis, we assessed the impact of Ct intervention on hepatic lipid deposition by histological analyses (Figure 5). The result of H&E staining revealed neatly arranged hepatocytes with no inflammatory cell infiltration in the NC group. Liver sections of the HFD group displayed a disarrangement in the architecture of hepatic cords with extensive intracellular vacuolization of hepatocytes, and the infiltration of inflammatory cells and accumulated red blood cells were visible. In comparison to the HFD group, the liver sections in the Ct-treated groups showed fewer lipid vacuoles and less inflammatory cell infiltration. Accordingly, 10^7^ CFU/mL Ct and 10^8^ CFU/mL Ct could reduce the degree of HFD-induced liver injury. The oil red O results demonstrated large quantities of lipid vacuoles and droplet deposition in the liver tissue samples of the HFD group, but the NC group did not exhibit evident histological changes, implying that chronic consumption of HFD can induce a certain degree of hepatic fat metabolism disorder and then lead to excessive fat deposition in the liver. Furthermore, the HFD + HC group had fewer fat vacuoles and red lipid droplets compared to the HFD + LC group, suggesting that Ct effectively relieved lipid accumulation in the liver of mice by altering these histological changes.

### 3.4. Effect of Ct on Liver Lipid Deposition in Obese Mice

**Figure 6:**
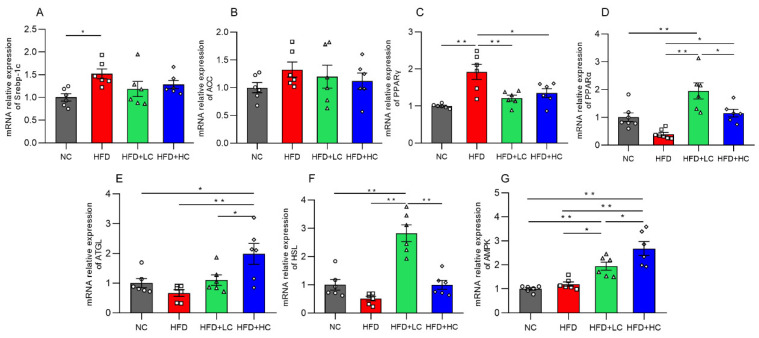
Impact of Ct on mRNA expression of genes linked to liver lipid metabolism. Relative mRNA expression of (**A**) Srebp-1c; (**B**) ACC; (**C**) PPARγ; (**D**) PPARα; (**E**) ATGL; (**F**) HSL; (**G**) AMPK. The gray bars and open circles (○) represent the NC group, red bars and open squares (□) represent the HFD group, green bars and open triangles (△) represent the HFD + LC group, and blue bars and open rhombus (◇) represent the HFD + HC group. Data are represented as mean ± SEM and one-way ANOVA was performed, *n* = 6–7, * *p* < 0.05, ** *p* < 0.01.

Genes connected to lipid metabolism were assessed for their levels of mRNA expression to further investigate the potential molecular mechanism of Ct in modulating hepatic lipid metabolism in mice (Figure 6). The mRNA expression of the lipogenesis-related gene Srebp-1c and the adipose differentiation-associated gene PPARγ was significantly elevated with HFD stimulation in comparison to untreated controls (*p* < 0.05, Figure 6A,C). Additionally, in the comparison between the HFD and NC groups, we found that there were no significant effects on the mRNA expression of the lipogenic gene ACC, fatty acid oxidation-related gene PPARα, lipolysis-related genes (HSL, ATGL), and the regulator of energy metabolism such as AMPK (*p* > 0.05, Figure 6B,D–G).

Interestingly, both the low- and high-dose *C. tyrobutyricum* treatment significantly downregulated the HFD-induced mRNA expression of PPARγ (*p* < 0.01, *p* < 0.05, respectively). However, AMPK, PPARα, and HSL mRNA levels were considerably higher in the HFD + LC group than in the HFD group. Similarly, there was an increase in the levels of AMPK, ATGL, and PPARα mRNA expression after the high concentration of Ct treatment. Thus, this suggested that Ct can regulate lipid metabolism.

### 3.5. Ct Alleviates HFD-Induced Intestinal Mucosal Injury

**Figure 7:**
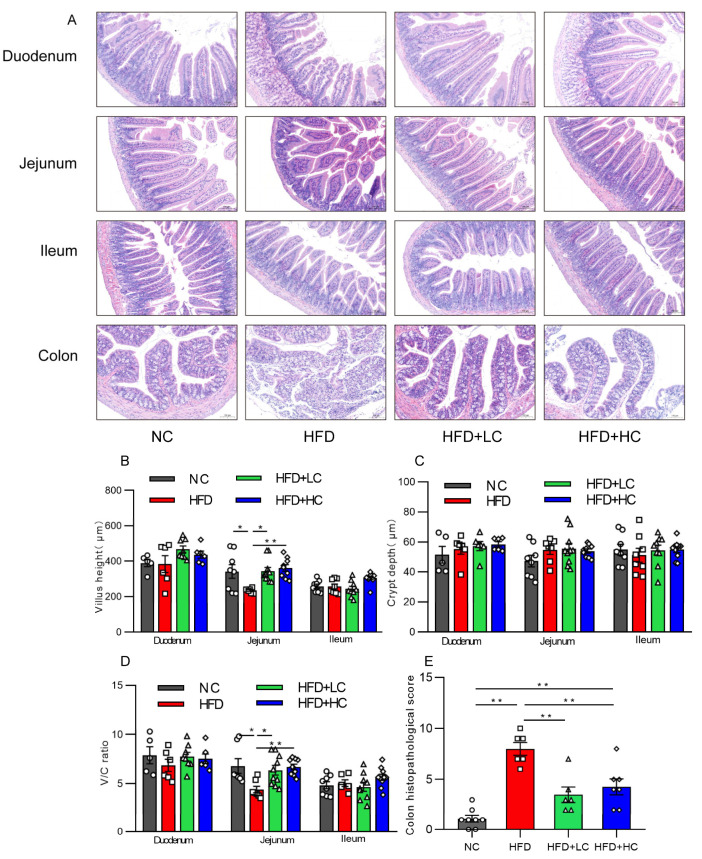
Impact of Ct on structures and morphology of intestinal mucosal in mice. (**A**) H&E staining (scale bar: 100 μm); (**B**) villus height; (**C**) villus height/crypt depth (V/C); (**D**) colon histopathological injury; (**E**) colon histopathological score. The gray bars and open circles (○) represent the NC group, red bars and open squares (□) represent the HFD group, green bars and open triangles (△) represent the HFD + LC group, and blue bars and open rhombus (◇) represent the HFD + HC group. Data are displayed as mean ± SEM and were analyzed using one-way ANOVA, *n* = 5–10. * *p* < 0.05, ** *p* < 0.01.

Intestinal mucosal is an essential part of the intestinal barrier. The effect of Ct treatment on intestinal mucosal morphology in each intestinal segment was assessed by H&E staining under an optical microscope, as shown in Figure 7. An intact intestinal segment mucosa, with neatly arranged villi and glands, was observed in the NC group, while mice treated with HFD showed severe jejunal and colonic mucosal damage characterized by severe epithelial architecture destruction, damaged crypts, and inflammatory cell infiltration (Figure 7A). Villus height and the V/C of the jejunum of mice fed HFD showed a substantial reduction compared with those fed NC (*p* < 0.05, Figure 7B,D). Interestingly, those indicators were elevated after a 10^8^CFU/mL Ct intervention (*p* < 0.01, Figure 7B,D). Likewise, the histological scores of intestinal injuries also suggested that Ct treatment played a protective role against HFD-induced colonic damage (*p* < 0.01, Figure 7E), suggesting that the long-term high-fat induction resulted in noticeable changes in jejunal and colonic mucosal morphology, but that Ct can alleviate the symptoms. However, the NC group and the experimental groups did not significantly vary in the morphology or histological index of the ileum or duodenum (*p* > 0.05, Figure 7).

### 3.6. Ct Intervention Attenuates Intestinal Inflammation

**Figure 8:**
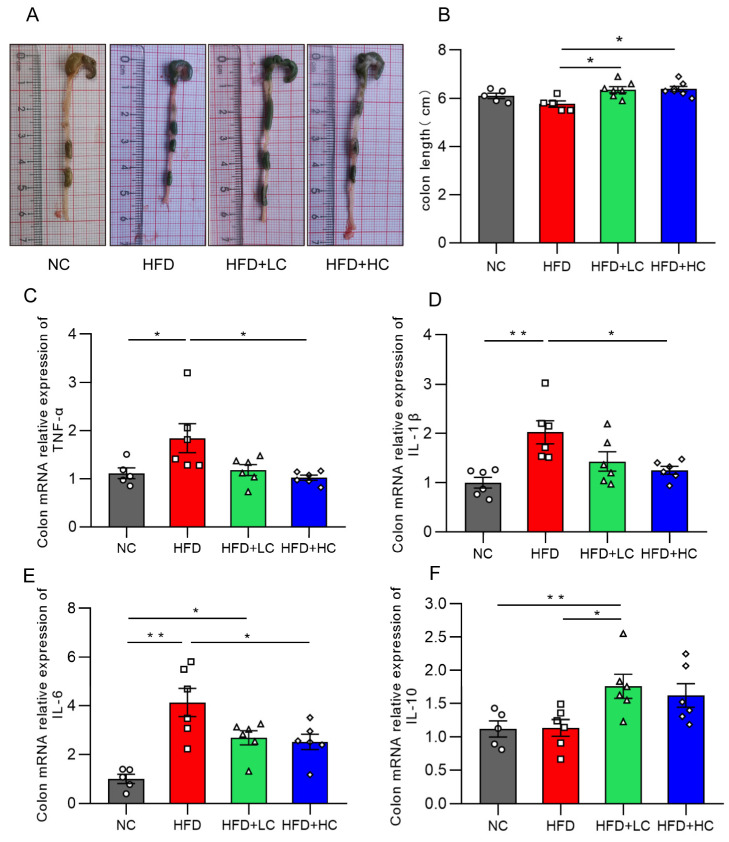
Effects of Ct on colon inflammation in mice. (**A**) Pictures of the colon; (**B**) colon length (cm); (**C**) mRNA expression of colon TNF-α; (**D**) mRNA expression of colon IL-1β; (**E**) mRNA expression of colon IL-6; (**F**) mRNA expression of colon IL-10. The gray bars and open circles (○) represent the NC group, red bars and open squares (□) represent the HFD group, green bars and open triangles (△) represent the HFD + LC group, and blue bars and open rhombus (◇) represent the HFD + HC group. Data are represented as mean ± SEM and were analyzed using one-way ANOVA, *n* = 5–7. * *p* < 0.05, ** *p* < 0.01.

Intestinal inflammation is a critical cause of intestinal barrier injury. Figure 8 illustrates that there was no statistically significant decrease in colon length between the HFD and NC groups (*p* = 0.2918, Figure 8B). This may be because the body size and weight in the control group were remarkably smaller. Notably, the *C. tyrobutyricum* group showed a significantly longer colon length than the HFD group (*p* < 0.05, Figure 8B).

The influences of Ct on the inflammatory cytokine mRNA expression levels in the colon are illustrated in Figure 8. We found that TNF-α, IL-1β, and IL-6 expression was substantially increased in the mice fed HFD for an extended period, as compared to the NC group. Strikingly, the HFD + LC group exhibited a substantial increase in the mRNA expression of IL-10 as compared to the HFD group (*p* < 0.05, Figure 8F). By contrast, the HFD + HC group revealed a notable decrease in TNF-α, IL-1β, and IL-6 mRNA levels of colon tissue (*p* < 0.05, Figure 8C–E). Overall, these results show that Ct can alleviate HFD-induced colonic inflammatory responses in mice.

### 3.7. mRNA Expression of Colonic Tight Junction Proteins

**Figure 9:**
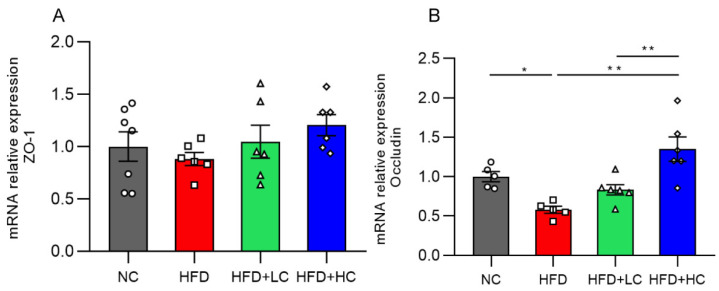
Effects of Ct on the barrier function of colon in mice. Relative mRNA expression of (**A**) Colon zonula occludens-1 (ZO-1); (**B**) colon Occludin. The gray bars and open circles (○) represent the NC group, red bars and open squares (□) represent the HFD group, green bars and open triangles (△) represent the HFD + LC group, and blue bars and open rhombus (◇) represent the HFD + HC group. Data are represented as mean ± SEM. One-way ANOVA was used to examine the significant distinction, *n* = 5–7, * *p* < 0.05, ** *p* < 0.01.

The impact of Ct supplementation on the mRNA expression of Occludin was assessed in the colon (Figure 9). Compared to the control group, the HFD group showed a marked decrease in Occludin mRNA levels in the colon tissues (*p* < 0.05, Figure 9B). However, there was a noticeable rise in Occludin mRNA expression in the colon of mice treated with Ct (10^8^ CFU/mL) (*p* < 0.01, Figure 9B). The above results proved that treatment with Ct can protect the intestinal barrier function in the colonic mucosa of obese mice.

### 3.8. Short-Chain Fatty Acid Concentrations

**Figure 10:**
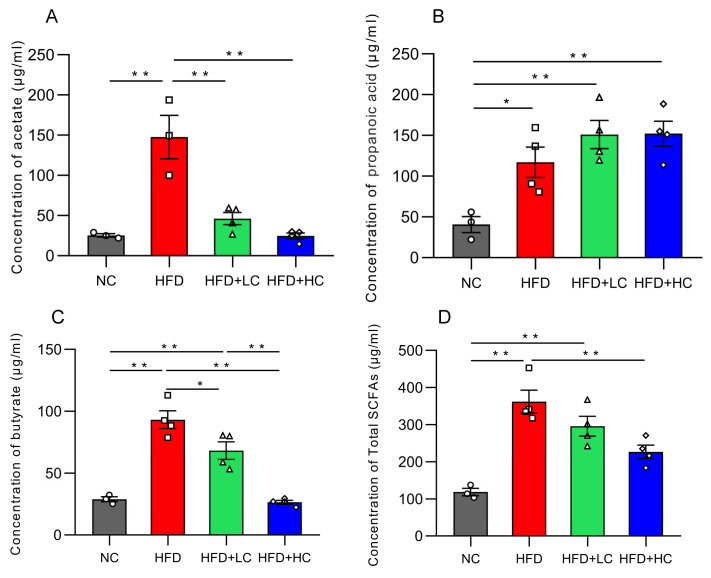
Effects of Ct on the concentration of SCFAs in colon content. (**A**) The concentration of acetate; (**B**) the concentration of propanoic; (**C**) the concentration of butyrate; (**D**) the concentration of total SCFAs. The gray bars and open circles (○) represent the NC group, red bars and open squares (□) represent the HFD group, green bars and open triangles (△) represent the HFD + LC group, and blue bars and open rhombus (◇) represent the HFD + HC group. Data are expressed as mean ± SEM and were analyzed using one-way ANOVA, *n* = 3–4. * *p* < 0.05, ** *p* < 0.01.

Acetic acid, propionic acid, butyric acid, and total SCFA concentration in colon contents dramatically increased in groups fed with a high-fat diet (*p* < 0.05, Figure 10). In contrast, the Ct-treated HFD-fed groups had considerably lower amounts of acetic acid, butyric acid, and total SCFAs than the HFD group (*p* < 0.05).

### 3.9. Ct Changes the Intestinal Microbiota Profile

Next, we explored the impacts of *C. tyrobutyricum* on the gut flora composition of HFD-induced mice. The microbiota were profiled in cecum samples by 16 S rRNA gene sequencing. According to our previous experimental results, the NC, HFD model, and HFD + HC groups were used for comparative analysis. A total of 1,021,180 high-quality sequences were obtained from all 18 samples from three groups, with a total length of 416,372,141 bp.

**Figure 11:**
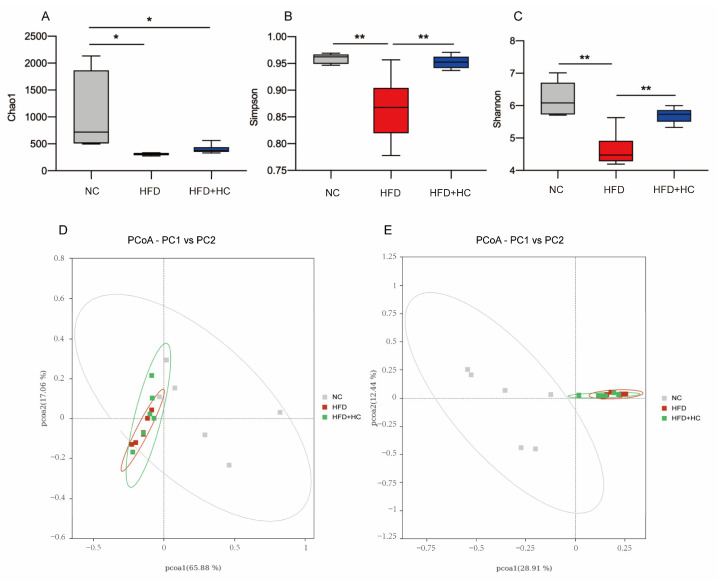
Alpha diversity and principal coordinates analysis of cecum content in mice. (**A**) Chao1 index; (**B**) Simpson index; (**C**) Shannon index; (**D**) PCoA analysis based on weighted Unifrac distance; (**E**) PCoA analysis ground on unweighted Unifrac distance. Data are represented as mean ± SEM and were analyzed using one-way ANOVA, *n* = 5–6. * *p* < 0.05, ** *p* < 0.01.

The richness and variety in samples are usually assessed using alpha diversity. Our study calculated the α-diversity using Chao1, Shannon, and Simpson indices. From Figure 11A, the microbial richness estimated by the Chao1 index demonstrated that the NC group had substantially greater richness than the HFD group and the HFD + HC group (*p* < 0.05). However, the Chao1 index of the HFD group did not differ significantly from the HFD + HC group (*p* = 0.9191). As compared to the NC group and the HFD + HC group, the Simpson and Shannon indexes were substantially lower in the HFD group (*p* < 0.01, Figure 11B,C). The above alpha diversity results suggested that a high-fat diet reduced the richness and diversity of intestinal flora in mice, but Ct intervention can mitigate this reduction.

Beta diversity metrics are used to assess the discrepancies in the makeup of microbial communities between samples. As shown in Figure 11D, principal coordinate analysis revealed that samples belonging to the same group had similar relationships. These groups were also relatively clustered together without significant partition. As shown in Figure 11E, PCoA based on Unweighted UniFrac distances found no clear zonation between the samples within the group. In addition, the HFD group and the HFD + HC group clustered relatively closely, whereas the distance between the NC group and the HFD group was relatively far (*p* < 0.05).

To sum up, we found that the microbial community structure within the group was highly similar. If we do not consider species abundance, there were significant differences in intestinal flora by long-term intake of HFD, yet the Ct intervention had no apparent effect on beta diversity.

### 3.10. Ct Improves HFD-Induced Gut Microbiota Disorder

**Figure 12:**
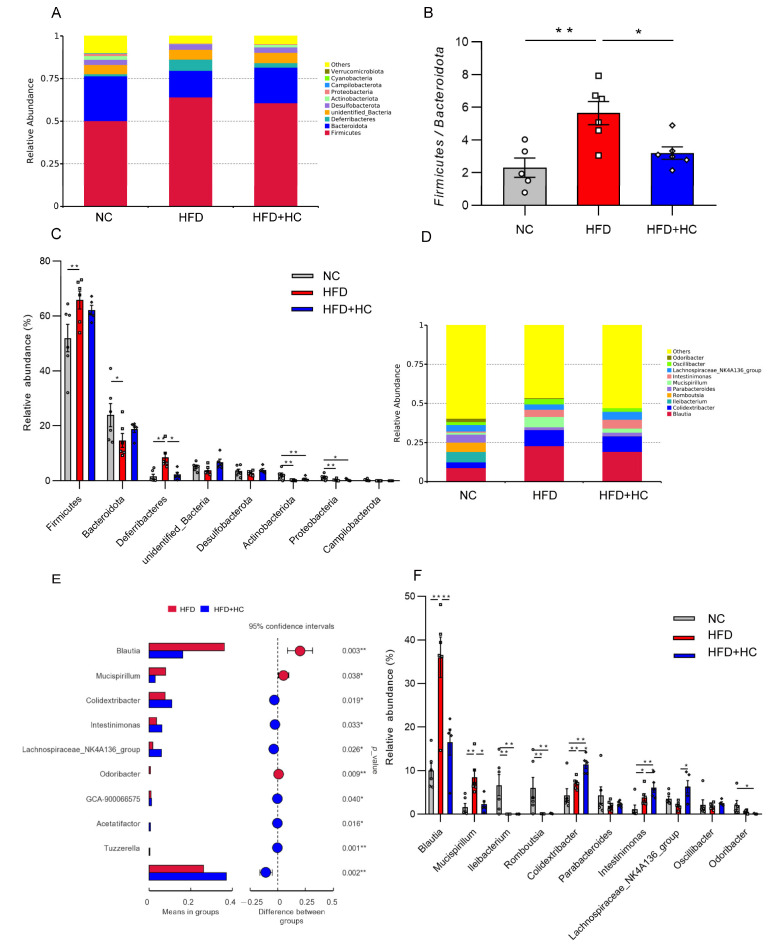
Relative abundance of microbiota in mice cecum content at the phylum and genus level. (**A**) Microbial community bar plot at phylum level; (**B**) the ratio of relative abundance of *Firmicutes* to *Bacteroidota*; (**C**) microbial differences at the phylum level; (**D**) microbial community bar plot at genus level; (**E**) microbial differences at genus level; (**F**) gut microbiota with significant difference between HFD group and HFD + HC group. The gray bars and open circles (○) represent the NC group, red bars and open squares (□) represent the HFD group, and blue bars and open rhombus (◇) represent the HFD + HC group. Data are represented as mean ± SEM and were analyzed using one-way ANOVA, *n* = 5–6, * *p* < 0.05, ** *p* < 0.01.

*Bacteroidetes* and *Firmicutes* were the most prevalent phyla among all dietary categories (Figure 12A). The *Firmicutes*/*Bacteroidetes* ratio was significantly increased in the HFD group (mean = 5.648) compared to the NC (mean = 2.313) and HFD + HC (mean = 3.204) groups (*p* < 0.05, Figure 12B). Then, the ten phyla with the highest relative abundance were further analyzed. The HFD group contained a higher proportion of *Firmicutes* and *Deferribacteres* relative to the NC group (*p* < 0.01, Figure 12C), while *Bacteroidota*, *Actinobacteriota*, and *Proteobacteria* were substantially reduced (*p* < 0.05). Compared to individuals with HFD intake, the gut microbiota of individuals with an intake of HFD plus high-dose Ct had a significantly lower abundance of *Deferribacteres* (*p* < 0.05).

*Blautia*, *Mucispirillum*, and *Colidextribacter* were substantially increased at the genus level in the HFD group, while *Ileibacterium* and *Romboutsiar* were substantially decreased in contrast to the NC group (*p* < 0.01, Figure 12F). The HFD + HC group exhibited a significant decrease in the relative abundance of *Blautia* and *Mucispirillum* as compared to the HFD group. In addition, high-dose Ct treatment resulted in a higher relative abundance of members of *Colidextribacter*, *Intestinimonas*, *Acetatifacter*, *Lachnospiraceae_NK4A136_group*, *GCA-900066575*, and *Tuzzerella* (*p* < 0.05, Figure 12E,F).

**Figure 13:**
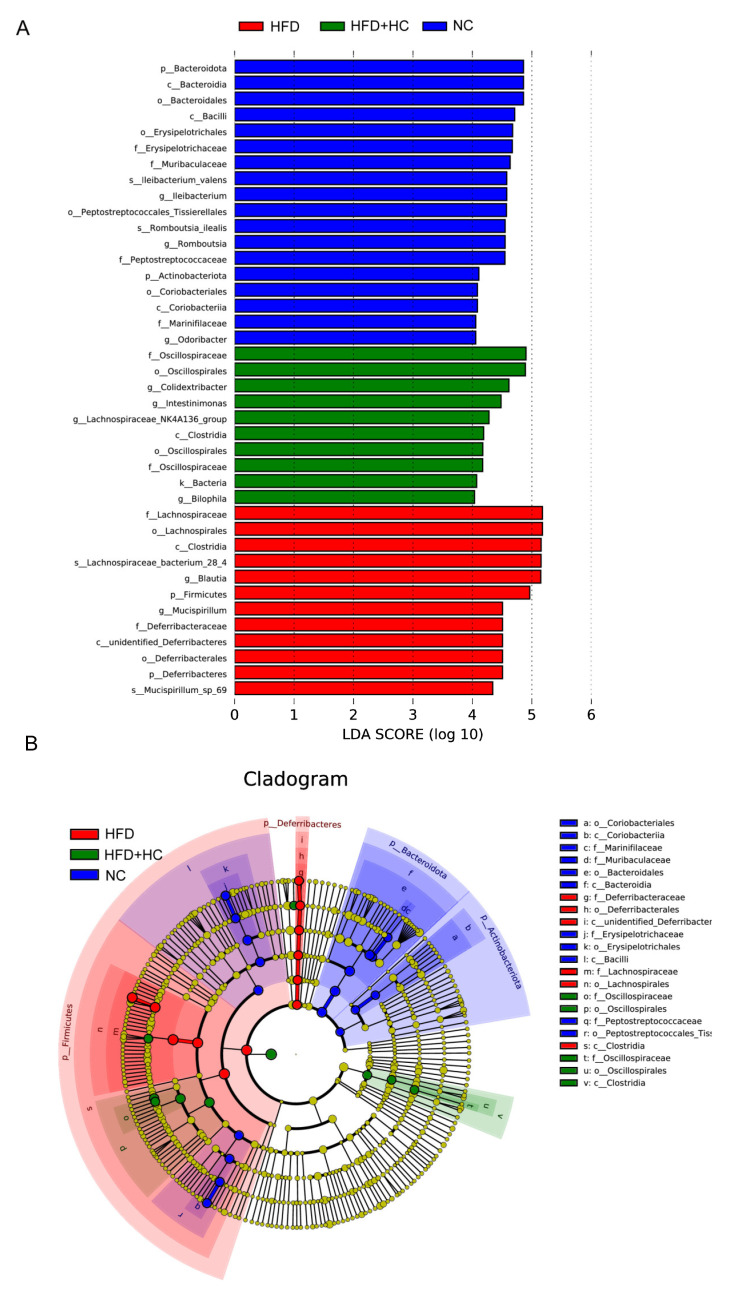
LEfSe analysis of microbiota in mice cecum content. (**A**) LEfSe analysis distribution histogram with an LDA score greater than 4. The red bars represent the HFD group, green bars represent the HFD + HC group, and blue bars represent the NC group; (**B**) cladogram generated from LEfSe analysis. Species with no significant differences are uniformly colored yellow. The red node indicates the microbial groups that play an important role in the HFD group, the green node indicates the microbial groups that play an important role in the HFD + HC group, and the blue node indicates the microbial groups that play an important role in the NC group.

When looking for flora that significantly varies in abundance between groups, one might apply the linear discriminant analysis effect size (LEfSe). We performed LEfSe analysis with an LDA score > 4 to further explore the Biomarker with statistical differences among all groups. Overall, 39 microbial groups were statistically different among the three groups (Figure 13). Of these, abundances of *Bacteroidota*, *Bacteroidia*, *Bacteroidales*, *Bacilli*, *Erysipelotrichales*, *Erysipelotrichaceae*, *Ileibacterium*, *Peptostreptococcaceae*, *Peptostreptococcales_tissierellales*, *Romboutsia*, *Actinobacteriota*, *Coriobacteriia*, *Marinifilaceae*, and *Odoribacter* had a significant impact on the NC group. The predominant species in the HFD group were *Firmicutes*, *Lachnospirales*, *Deferribacteres*, *Deferribacterales*, *Lachnospiraceae*, *Blautia*, *Mucispirrillum*, and *Mucispirrillum_sp_69.* Furthermore, the dominant species in the high-dose Ct intervention group were *Oscillospiraceae*, *Oscillospirales*, *Clostridia*, *Colidextribacter*, *Intestinimonas*, *Lachnospiraceae_NK4A136_group*, and *Bilophila.* Together, these results suggested that Ct intervention could partly remodel the microflora structure of mice.

## 4. Discussion

All over the world, obesity is becoming more common [2]. While the energy intake level exceeds the energy expenditure level, the energy storage spills over from adipose tissue and deposits in the muscle and liver, causing ectopic lipid deposition [28]. These tissues further respond by triggering an inflammatory response [29]. Obesity can cause liver abnormalities as well as a variety of comorbidities, such as cardiovascular diseases, which need to be properly addressed [5,6]. Probiotic supplementation appears to be an effective adjuvant therapy for obese people [19,20]. Our previous study on *C. tyrobutyricum* revealed its considerable high gastrointestinal tolerance and anti-inflammatory effect. Given that Ct was also linked to modifications in the expression of genes associated with hepatic lipid metabolism, we suspect that Ct treatment could alleviate obesity [24]. The present study offered novel evidence regarding the significant mitigating effect of *C. tyrobutyricum* intervention in lipid metabolic disorders brought on by the high-fat diet.

Among them, body weight is the most intuitive indicator for evaluating obesity. Indeed, we demonstrate that after receiving Ct treatment, the body weight of HFD mice noticeably decreased. The effect was even more prominent as the dosage increased. The indexes of serum lipid metabolism further supported this. High concentrations of triglycerides (TG), along with slightly or normally elevated LDL-C and increased small dense LDL, are typical dyslipidemias of obesity [30]. Our biochemical results indicate that a long-term intake of HFD can lead to lipid accumulation in serum, which can be significantly relieved by decreasing TG and LDL-C content after Ct intervention. Furthermore, we showed that the NC group had a lower HDL-C concentration than the HFD group, which could possibly be attributed to the lower amount of cholesterol surrounding the tissues in the NC group. Traditionally, HDL-C is essential for the transfer of reverse cholesterol. Extra cholesterol is transported by HDL-C from peripheral tissues to the liver for elimination or recycling [31]. Thus, our results indicated that improved lipid metabolism after Ct intervention was responsible for the markedly greater weight loss, rather than other reasons. To investigate the indicators of lipid deposition in the liver further, we performed the following experiments.

The liver is a vital organ in lipid metabolism. Because of an imbalance of lipid biosynthesis and decomposition, excess fat accumulation in the liver results in an enlarged liver [18]. In our study, the liver weight and liver index revealed a falling trend in the Ct group, and the outcomes of oil Red *O* and H&E staining confirmed the mitigating counterbalance effect of Ct on the hepatic lipid deposition, and high-dose Ct showed a more potent effect. Our data have further revealed that HFD causes lipid accumulation in the liver, whereas the liver weight, TC, TG, and NEFA contents dramatically decreased after Ct treatment. Thus, while further investigations about the Ct intervention mechanism are needed, it is clear that Ct could inhibit lipid deposition.

The vital nutrient sensor AMPK is involved in metabolism and in regulating the energy equilibrium of the entire body [32]. In the liver, the activation of AMPK leads to an increased oxidation of fatty acids and reduced synthesis of triglycerides and cholesterol [33,34]. Also, ATGL and HSL are key rate-limiting enzymes in lipolysis. In mice, systemic or hepato-specific ATGL loss has been shown to cause adipose lesions in liver tissue [13]. In humans, HSL mutation can result in a reduced capacity for lipid storage, and the importance of HSL-mediated lipolysis in cellular signaling processes was displayed [14]. In addition, AMPK also upregulates the expression of PPARα, which is vital for the liver’s fatty acid absorption and β-oxidation [35]. SREBP1c is a central transcription activator of fatty acid biosynthesis, which induces hepatic steatosis by increasing TG accumulation [16]. Overexpression of PPARγ induces the development of fatty livers by upregulating the expression of SREBP1c expression. To shed more light on the function of Ct in the hepatic lipid metabolism, we analyzed the hepatic mRNA levels of lipid-metabolism-related genes. Our findings align with these previous reports and highlight that Ct intervention reduced lipid deposition in the liver by downregulating PPARγ expression and upregulating AMPK, ATGL, HSL, and PPARα expression. This validates the results of our previous transcriptomic data and is consistent with the research of Kershaw et al. [24,36].

Obesity is a major source of chronic and systemic inflammation [37]. In animal models, numerous studies have shown that elevated expression of certain proinflammatory cytokines or indicators of inflammation is linked to diet-induced obesity. Ding et al. (2010) reported that HFD feeding could induce immunological disorders and promote low-grade inflammation [38]. Previously, Fu et al. reported that butyrate may be able to lessen the degree of inflammation in the colon and ileum [39,40]. As is well known, Ct is a butyrate-producing bacterium. Thus, we characterized several inflammatory markers and discovered that the increase in TNF-α, IL-1β, and IL-6 mRNA concentrations after high-fat feeding was significantly abolished by the Ct treatment, while IL-10 expression was noticeably higher in the HFD + LC group. In addition, the colon lengths of the mice in different Ct treatment groups increased, further confirming that Ct effectively decreases HFD-induced murine colonic inflammation. Obesity, on the other hand, has been linked to disruption in the intestinal barrier function; so, we assume the inflammation was accompanied by the damaged intestinal structure [41].

The indexes for assessing intestinal health include intestinal crypt depth, villus height (VH), and villi height/crypt depth ratios [42]. Indeed, our data show that chronic consumption of an HFD by mice led to evident changes in the jejunum mucosa morphology and decreased mRNA expression of tight junction protein Occludin in the colon. Some research has demonstrated that certain commensals and probiotics can halt or even reverse the negative effects of pathogen infections on the function of the intestinal barrier [43]. Colonic mucosa thickness was markedly enhanced by *C. butyricum* Sx-01. Its ability to produce butyrate could account for the impact of *C. butyricum* on mucosal health [44]. Of note, butyrate is known to have a mucosal protective role in the gut, and research by Peng et al. (2009) showed that butyrate strengthens the intestinal barriers by facilitating the dynamic process of tight junction formation, which is regulated by AMPK activation [45]. Here, we demonstrate that Ct intervention can effectively improve the jejunal villus injury induced by HFD and has a specific repair effect on colonic damage. Among them, Ct dosage at 10^8^ CFU/mL could better regulate the expression of Occluding in colonic tissues, suggesting that Ct enhances intestinal barrier function by upregulating tight junction protein expression.

As a critical ‘endocrine organ’, the intestinal flora can promote the development of obesity through various mechanisms [18]. Previous research has demonstrated a link between obesity and a change in intestinal microbiota [46,47]. On a long-term basis, HFD can reduce both the variety and abundance of the cecal and fecal microbiome [48,49]. In 2004, Gordon and colleagues [50] first reported that the intestinal flora could regulate lipid metabolism by downregulating the expression of lipid oxidation genes and upregulating the expression of lipogenic genes, thereby leading to excessive fat deposition in mice. They then inferred that the intestinal flora is essential to inducing obesity via experiments in germ-free mice. Some studies have shown a strain-specific relationship between the intestinal microbiota and obesity development. Disregarding kinship and in comparison to lean mice, *Bacteroidetes* are 50% less common in obese mice, while *Firmicutes* are proportionately more prevalent [51]. The study by Tomas et al. showed that obesogenic diets drastically altered the composition of microbiota, resulting in a profile marked by a drop in *Bacteroidetes* and *Candidatus Arthromitus* as well as an expansion in *Firmicutes* (appearance of *Erysipelotrichi*) and *Proteobacteria*. Currently, the research for intestinal microflora has become the new frontier in the development of obesity. Here, we used 16S rRNA gene (V3-V4) sequencing to determine that 12-week HFD consumption affects the makeup of the cecal microbiota, as previously shown, perhaps best evidenced by the highly significant difference in α- and β- diversity. The Chao1, Simpson, and Shannon diversity indexes were decreased in the HFD-challenged group, indicating lower bacterial diversity in obese mice, but Ct intervention appears to mitigate the adverse effect. In obese mice, it has been proposed that a higher F/B ratio is a common sign [52]. When we specifically analyzed the microbiota associated with the cecal contents, we found that *Firmicutes* and *Bacteroidota* dominated the intestinal microbiota of all groups at the phylum level, and the HFD mice had a noticeably higher F/B ratio compared to the NC group and HFD + HC group. Collectively, Ct intervention could improve the metabolic disorder of intestinal flora induced by HFD to a certain extent.

Other bacterial species were affected under HFD. Further analysis of differential genera showed that HFD favored the emergence of the genus *Blautia*, *Mucispirillum*, *and Colidextribacter*, in contrast to that observed in the NC group, a conclusion backed by comparable findings in mouse models. *Blautia*, in particular, has been linked to disorders of physiological function such as obesity, type 2 diabetes, and inflammatory diseases as a gut microbiota marker for obesity [53,54]. Zhang and colleagues demonstrated a striking increase in *Mucispirillum* in NAFLD mouse models, accompanied by lipid deposition and liver inflammation [55]. Additionally, Spearman’s rank correlation analysis implied that *Colidextribacter* was robustly associated with particular bile acid monomers and positively correlated with oxidative stress [56]. Notably, treatment with Ct significantly improved these HFD-induced changes. As our data shows, *Bilophila*, *Intestinimonas*, and *Lachnospiraceae_NK4A136_group* were predominant bacteria genera in the cecum of the HFD + HC group. Among them, *Bilophila* is a bile-tolerant microorganism. *Intestinimonas* is generally considered a beneficial genus with anti-obesity characteristics because of its butyrate-producing ability [57]. *Lachnospiraceae_NK4A136_group* can produce SCFAs, including propionic and butyric acid, which have been linked to enhancing the gut barrier function [58].

Lately, the contribution of SCFA to obesity has received attention. Among them, mammalian lipid metabolism requires the substrates acetate and butyrate, while gut-derived propionate is a major factor in gluconeogenesis [59]. In our investigation, elevated levels of SCFAs were noticed in the colonic contents of high-fat diet-fed mice. Interestingly, although Ct is a butyrate-producing bacterium for our therapeutic intervention, the highest butyric acid level was in the HFD group. We presume that there might be two reasons for this contradictory finding. First, these increased SCFAs may originate from the breakdown of lipids in HFD. Second, many *Firmicutes* are butyrate producers, and the number of *Firmicutes* increased in obese mice, which was significantly higher than that observed with Ct gavage [60,61,62]. On the genus level, the rising level of acetate in the HFD group might be ascribed to the distinguished increase in *Blautia*, which was reportedly involved in acetate and butyrate production [63,64,65]. *Lachnospirillaceae*, a prominent bacterial family in the mammalian gut, was found to dominate within the HFD group according to the LEfSe analysis. *Roseburia* and *Eubacterium* genera are two well-known butyric acid producers in the *Lachnospiraceae* family [66]. Based on this observation, we hypothesized that the cecal concentration of butyric acid significantly increased, possibly caused by the stimulation of *Lachnospiraceae*. In agreement with this, according to research on mice, the obese microbiota produces extra SCFA, which increases colonic energy availability and may lead to obesity. Some fat people have also been observed to have higher fecal SCFA concentrations than lean humans [67,68]. However, we have demonstrated a significant decrease in straight SCFA levels after Ct intervention, including acetate and butyrate. Overall, our findings suggest that Ct may reduce metabolic risk factors linked to obese people’s elevated colonic SCFA levels. A limitation of our study is that our analyses of SCFAs were confined to the mouse colon contents but not whole-body SCFA metabolism. Further research is needed to determine the specific mechanisms that would account for the increased colonic SCFA concentrations in HFD mice.

To sum up, our results suggest that *Clostridium tyrobutyricum* could ameliorate the lipid metabolism issue brought on by HFD, maintain the integrity of the intestinal barrier, and modulate intestinal microbiota structure. These results offer a novel perspective into the role of Ct as a probiotic in the regulation of lipid metabolism by modulating intestinal health.

## 5. Conclusions

*Clostridium tyrobutyricum* administration can reduce serum and liver lipid deposition in mice and alleviate HFD-induced body and intestinal inflammatory responses. In addition, Ct can also improve intestinal morphological damage, and increase the number of beneficial bacteria in the intestine, which has the role of maintaining intestinal health and has a good application prospect.

## Figures and Tables

**Table 1:** The diets’ ingredients and nutritional makeup.

Nutrient Class	Ingredient	General Diet (gm)	High-Fat Diet (gm)
Protein	Casein	200	200
	L-Cystine	3	3
Carbohydrate	Corn Starch	506.2	0
	Maltodextrin 10	125	125
	Sucrose	72.8	72.8
Fiber	Cellulose	50	50
Fat	Soybean oil	25	25
	Lard	20	245
Mineral	Mineral Mix S10026B	50	50
Vitamin	Vitamin Mix V10001C	1	1
	Choline Bitartrate	2	2

**Table 2:** Evaluation criteria of histological damage of the colon.

Feature Graded	Description	Grade
Normal tissue	None	0
Degree of epithelial surface damage	Local and slight	1
Degree of Crypt damage	Local and moderate	2
Degree of inflammatory factor infiltration	Local and severe	3
	Extensive and moderate	4
	Extensive and severe	5

**Table 3:** The primers of RT-qPCR in liver tissue.

Genes	Forward Primer	Reverse Primer
ACC	TGTCCGCACTGACTGTAACCA	TGCTCCGCACAGATTCTTCA
Srebp-1c	GCCATCGACTACATCCGCTTCTTG	TGCCTCCTCCACTGCCACAAG
AMPK	TGAAGATCGGCC ACTACATCCT	CTTGCCCACCTTCACTTTCC
PPARα	CAGGAGAGCAGGGATTTGCA	CCTACGCTCAGCCCTCTTCAT
PPARγ	AGGGCGATCTTGACAGGAAAGAC	AAATTCGGATGGCCACCTCTTTGC
HSL	CAGGAGAGCAGGGATTTGCA	CCTACGCTCAGCCCTCTTCAT
ATGL	CGCGCTCTTGGCTCATG	CCAACCTTTGTGCCCCTTAA
β-actin	CGTTGACATCCGTAAAGACC	AACAGTCCGCCTAGAAGCAC

**Table 4:** The primers of RT-qPCR in colonic tissue.

Genes	Forward Primer	Reverse Primer
TNF-α	GCGACGTGGAACTGGCAGAAG	GAATGAGAAGAGGCTGAGACATAGGC
IL-1β	TGCCACCTTTTGACAGTGATG	ATGTGCTGCTGCGAGATTTG
IL-6	AGACTTCCATCCAGTTGCCTTCTTG	CATGTGTAATTAAGCCTCCGACTTGTG
IL-10	CCAAGCCTTATCGGAAATGA	TCCTGAGGGTCTTCAGCTTC
ZO-1	AGGACACCAAAGCATGTGAG	GGAGATTCCTCTGACCTTGAGTGT
Occludin	GGAGATTCCTCTGACCTTGAGTGT	TTCCTGCTTTCCCCTTCGT

## Data Availability

The corresponding author can be contacted for access to the data that support this study’s conclusions.
